# New Approach for Securing and Dating Valuable Printed Documents

**DOI:** 10.1002/gch2.201800097

**Published:** 2019-05-28

**Authors:** Altaf H. Basta, Houssni El‐Saied, Ahmad S. Salim, Mohamed A. Mohamed

**Affiliations:** ^1^ Cellulose and Paper Department National Research Centre Dokki Giza 12622 Egypt; ^2^ Forgery Research Department Medico‐legal Sector Ministry of Justice Cairo 11461 Egypt

**Keywords:** counterfeiting and forging tests, dating documents, fluorescence compounds, forging crime detection on valuable documents, machine identification code (MIC), security paper documents, UV inks

## Abstract

This work deals with using a novel approach for securing and dating printed documents, which will detect any forging crime present. In this respect, the coded dots matrices (machine identification code (MIC)) for the used printers are extracted via a binary system of base 2 (0, 1). The UV ink used in the printing process is prepared from novel fluorescence compounds together with polyvinyl alcohol. Different Xerox models of color laser printers are used for printing the document designs. The data obtained reveal that the investigated approach is succeeded in specifying the date of the printing process together with translating the embedded data of the printer to visible information, which can be tracked under a UV lamp. This innovative approach will assist workers in examining the questioned document by specifying the authorized date and position of printed documents from the MIC.

## 1 Introduction

In recent years, attention has been focused on developing and enhancing the quality and safety performance of valuable paper documents (checks, receipts, etc.). Many investigators studied the role of paper sheet compositions (type cellulosic fibers and sizing agent), metal complexes, fire retardant additives, coating by biopolymers, as well as surrounding environment on the quality and durability of paper.^1^, ^2^, ^3^, ^4^, ^5^, ^6^, ^7^, ^8^ While, for forging purposes, safety paper must be durable and include safety marker resistance to forgery and counterfeiting.^9^, ^10^, ^11^, ^12^

Fluorescence active compounds are important materials succeeded to gain the paper document safety property. In this subject, we synthesized 2‐alkoxy‐3‐pyridinecarbonitrile derivatives as well as heterocyclic compounds gathering the whole functional moieties responsible for fluorescence properties. In the synthesis we used variety of pyridine derivatives possessing both amino and alkoxy group oriented o‐ and o′‐positions of the pyridine nucleus and neighboring to nitrile functions. The behavior of these compounds in nanoparticles form as security marker for production of unfalsifiable documents by erasure technique (chemical and mechanical) was also studied.^9^, ^10^, ^12^

On the other hand, the determination of the document date is a very necessary query in the field of the questioned document examination. It is also one of the most challenging and dialectical issues. Numerous approaches have been developed to address this issue. The first approach is “time tags”, which relates the concomitant properties of the ink and the paper via the introduction date of paper substrate, or market ink. The second approach is used to investigate the aging of documents, but unfortunately it is not influenced only by the passage time but also by the storage conditions and the document components. Finally, the third approach is focused on the relative age of the documents and aims at reconstructing their chronology.^13^ In general, the application of these approaches is complex and accompanied with many drawbacks. In digital forensic labs, there is a serious dilemma that is concerned with determining the date of the documents. In the recent decade via developing the technology of color laser printing, we could do a little bit to achieve efficient results in determining the date of the questioned documents, using the special types of color laser printing.

The work of the active technique aims for tracking the extrinsic features embedded in the colored machines, on the contrary with the passive technique that focused on using the imperfection raised from optical, electrical, or mechanical defects in the machines.^14^ The form of active technique appears as yellow dots, which are called machine identification codes (MIC) and counterfeit protection system (CPS) codes.^15^, ^16^, ^17^ The tracking dots sizes are very small and cannot be seen via the naked eye.^18^, ^19^, ^20^ So, the tracking of these yellow dots will guide to information about the color laser machines such as serial number, model, as well as, in special cases, the time and date of the printing process. From this point of view and for enhancing the application of fluorescence heterocyclic compounds, in this present work we evaluate the role of fluorescence active fluorinated pyrazolines as active compounds for extracting the MIC and securing together with dating the valuable printed documents.

Recently, the authors succeeded in synthesizing a variety of fluorescence‐active fluorinated pyrazolines (21 compounds) in good yields through cyclocondensation reaction of propenones with aryl hydrazines. The synthesized compounds, which have promising fluorescence properties (quantum yield (*Ф*s) reached 0.86 with respect to quinine sulfate),^21^ are candidates in this present work to formulate the UV ink together with polyvinyl alcohol (PVA), as a novel application to translate the embedded data and extract the coded dots matrices (MIC) from the used printer to visible, which can be tracked under the UV lamp. This will serve to detect any forgery present in valuable documents.

## 2 Results and Discussion

### 2.1 UV–Vis Absorption and Fluorescence Spectra

The UV–vis spectra of the synthesized pyrazolines 7–10 were estimated in chloroform with constant concentration (4 mg L^−1^). The maximum absorption wavelength (λ_max_) and molar extinction coefficient (ε_max_) are recorded in **Table** **1**. It is clear that all investigated pyrazolines have two prominent peaks around 238 and 358 nm, which are related to the π–π* and n–π* transitions, respectively. The effect of introducing different electron‐donating (EDG) and withdrawing groups (EWG) in R, R′, and R″ have a remarkable effect on the differences in both the position and intensity of absorption peaks, whereas, the presence of the 2‐naphthyl group in compound 9 provided an additional adsorption peak together with a higher absorption peak relative to the other synthesized analogues.

**Table 1:** Absorption, excitation, and emission spectral properties of active fluorescence pyrazoline compounds (7–10) in chloroform

Fluorescence active pyrazolines	Absorption		Fluorescent measurements of active pyrazolines		
	λ_max_ [nm]	ε_max_	Excitation λ_max_ [nm]	Emission λ_max_ [nm]	*Φ*_s_
7	241	117 001.805	267	455.8	0.819
	367	67 096.2375	369.3^a)^		
8	242	45 431.375	265	443.7	0.858
	358	37 171.125	363.2^a)^		
9	243	189 520.7475	294	453.7	0.553
	277	116 558.7675			
	375	83 685.7875	373.7^a)^		
10	242	104 819.485	300	471.6	0.844

^a)^ Wavelength used in the calculation of fluorescence quantum yield of compounds 7–10.

With regard to the fluorescence spectra of synthesized pyrazolines, this test is also undertaken in chloroform with constant concentration (1 × 10^−5^ mol L^−1^) (Table 1). Table 1 shows that the synthesized pyrazolines are excited at 360–370 nm (corresponding to the high wavelength absorption band), affording fluorescence emission in the blue to green regions (emission peak wavelengths 438–471 nm). It has also been noticed that the quantum yield (*Ф*_sample_) value is greatly affected by the substitution‐type synthesized pyrazolines. The fluorescence quantum yields were measured relative quinine sulfate using the following equation:

(1) $$\emptyset_{\mathrm{sample}}=\emptyset_{\mathrm{ref}}\left(\frac{F_{\mathrm{sample}}}{F_{\mathrm{ref}}}\right)\left(\frac{A_{\mathrm{ref}}}{A_{\mathrm{sample}}}\right)\left(\frac{\eta_{\mathrm{sample}}^{2}}{\eta_{\mathrm{ref}}^{2}}\right)$$

where *A* = absorbance, *F* = fluorescence, *F*_sample_, *A*_sample_, η_sample_, and *F*_ref_, *A*_ref_, η_ref_ are relative integrated fluorescence intensities, absorbance at excitation wavelength, and refractive index of the sample and reference, respectively.

It can be seen that the presence of EWG and bulky groups in R′, such as those present in compounds 7 and 10, a higher quantum yield with blue‐shifting of emission spectra than the other synthesized compounds occurs.

On applying these fluorescence compounds to treat the Bible paper sheets, **Table** **2** shows that the treated paper sheets are excited at the range of 360–370 nm that corresponding to the absorption band in the solutions. It is noticed that the presence of the 4‐chlorophenyl group at pyrazoline moiety for compounds 7 and 9 provides higher intensity; however the lower intensity is illustrated in case of treated paper with compounds including 4‐methylphenyl, as the case of compounds 8 and 10.

**Table 2:** Fluorescence spectra of treated paper sheets

Sample no.	Excitation	Emission	Fluorescence intensity
	maxima λ_max_ [nm]	maxima λ_max_ [nm]	
Untreated Paper	–	–	–
Treated with Cpd. 7	279.5, 399.9	467.7	3077.900
Treated with Cpd. 8	279.7, 380	453.2	1688.620
Treated with Cpd. 9	281371	458.3	4755.480
Treated with Cpd. 10	280, 376	467.6	1360.270

### 2.2 Fluorescence Compounds as Secure and Dating Markers

To evaluate the role of the investigated approach for translating the machine identification codes to date the printed valuable documents, four fluorescence compounds (7, 8, 9, and 10) with different fluorescence quantum yield *ф*s (0.55–0.86) were used to prepare UV inks. We designed two forms of check and receipt by using Microsoft Office 2016. Then we printed the designed forms on the paper sheets using different color laser printers of Xerox brand. The safety performance of the valuable documents is achieved by the following stages.

#### 2.2.1 Extracting the Authorized Data from Printer

**Figure 1:**
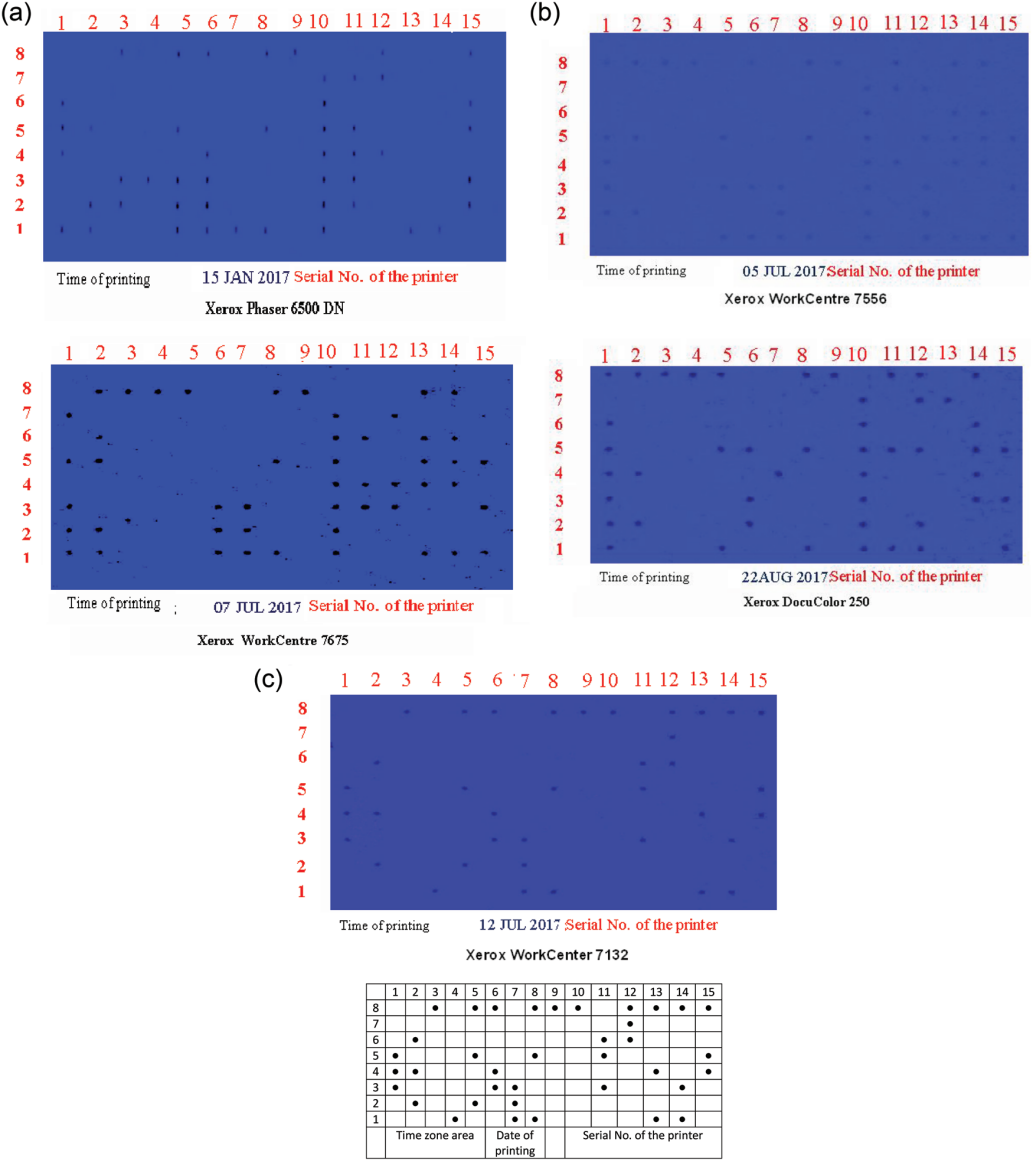
a) Exhibiting the machine identification code (MIC) of different Xerox laser printers, with the date of the printing process. b) Exhibiting the machine identification code (MIC) of different Xerox laser printers, with the date of the printing process. c) Exhibiting the simulated machine identification code related of Xerox WorkCenter 7132.

The investigated approach is applied for extracting the authorized data printed from five color laser printers (Xerox Phaser 6500 DN, Xerox WorkCentre 7675, Xerox WorkCentre 7556, Xerox DocuColor 250, and Xerox WorkCenter 7132). This approach includes scanning of the printed paper sheets at 1200 dots per inch (DPI). Adobe Photoshop CC 2015 was used as an image processing technique for the extraction step. Finally, we extracted all the machine identification codes that are printed on paper sheets. **Figure** **1**a–d illustrates the distributions of MIC for each type of printer used. It is clear that the authorized data printed on papers are presented in a form of binary system dots that are machine known. Based on this the authorized data are calculated and converted from binary system state to decimal numbers.

From Figure 1a–c, it is observed that all the coded dots matrices (MIC) for the used color lasers consist of a regular grid of dots spread in fifteen columns and eight rows in a defined pattern, and then the area that exhibited the date of the printing is specified. There are three columns numbered 6, 7, and 8 that express the day, month, and year of the printing process, respectively. The date of the printing could simply be calculated from the binary system for base 2 (0, 1), and the calculation direction from down to up. The extraction and the calculation of the date of the printing process are as follows:

* For the unit of the year as present in Figure 1a–c, in row number one and column number eight, one point is found that is calculated as 2^0^ and equals one. At the same column but at row number five, it contains another dot that calculated as 2^4^, which is equivalent to sixteen. The specified year 2017 is a result of summation.
* From Figure 1b, which is corresponds to the Xerox DocuColor 250 printer, at column seven and row number four, one point only is observed, which is calculated as 2^3^ and is equivalent eight. This is the month of printing.
* For Xerox WorkCenter 7132 (Figure 1c), from column number six, it contains two dots at rows number three and four that calculated as 2^2^ and 2^3^. The summation represents the day of the printing.

#### 2.2.2 Illustrating the Extracted Date on Printed Paper Documents

**Figure 2:**
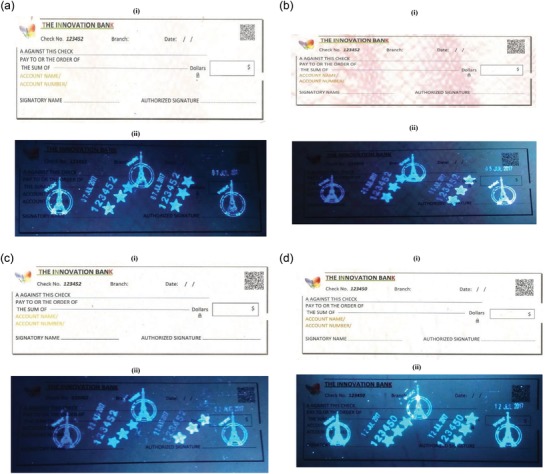
a) Designed check printed with fluorescence compound 7‐based UV ink (i) and under UV light at 365 nm (ii). b) Designed check printed with fluorescence compound 8‐based UV ink (i), and under UV light at 365 nm (ii). c) Designed check printed with fluorescence compound 9‐based UV ink (i), and under UV light at 365 nm (ii). d) Designed check printed with fluorescence compound 10‐based UV ink (i) and under the UV light at 365 nm (ii).

**Figure 3:**
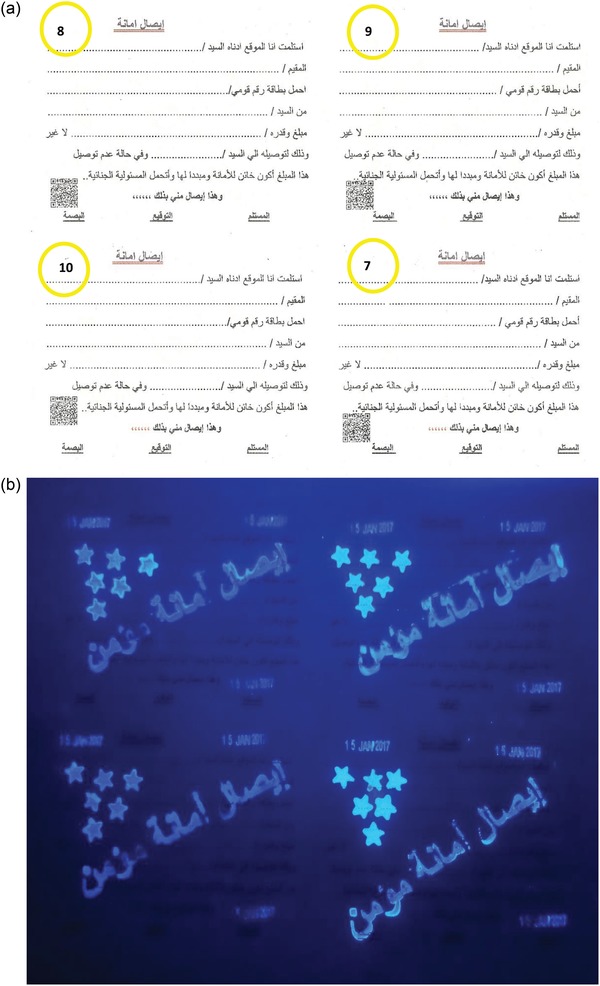
a) Live image of the designed receipt printed with the four UV‐ink‐containing compounds 7, 8, 9, and 10, as indicated in yellow circles. b) UV images of the four receipt printed from UV inks made from fluorescence compounds 7–10.

The extracted and calculated dates are clear on the valuable document by using the prepared UV via print dater, numerator, and stamp print, using Sirdas print dater 5810, numerator, and stamp print design, respectively. The used UV inks from four selected novel compounds (7, 8, 9, and 10) are colorless. The final images of the designed checks and receipt, under UV light at 365 nm are given in **Figures** **2**a–d and **3**.

**Figure 4:**
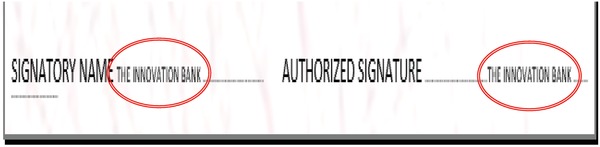
Indication of the micro test created in the designed check model and magnified inside red two ovals.

**Figure 5:**
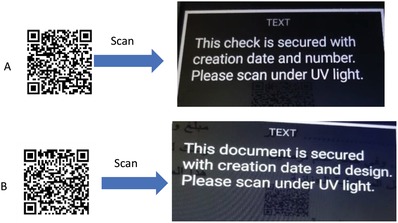
Exhibiting two static QR and their included text after scan: A) for check and B) for honest rest.

With regard to designed check model and for improving the safety performance of paper documents, we have created the printed number of the check and micro text in two positions adjacent to two sentences “SIGNATORY NAME” and “AUTHORIZED SIGNATURE.” By magnifying at 20× it is possible to read the micro text sentence “THE INNOVATIVE BANK,” as given in **Figure** **4**. Moreover, a new feature added at the upper right corner of the check and down left of receipt is quick respond barcode (QR). This added barcode is a static one, and it is just scanned with any barcode reader to benefit the worker in a bank or in the field of the questioned document examination, through the text messages appeared for both check and receipt as clear in **Figure** **5**.

With regard to check design (Figure 2a–d), the number of the check is printed adjacent to the text “Check No.” It is printed with two hidden numbers, using colorless synthesized UV inks, while, the date of the printing process or the creation date of the document is printed in three different positions in both check and receipt with the invisible UV inks. The Eiffel Tower of Paris is also printed as a latent image. Finally, we printed six stars to complete the design for both checks and receipt in different positions with the invisible UV inks to promote the document security and difficulty to forgery. These printed stars indicate

* Presence of secured star
* Presence of micro print
* Presence of fluorescent number of novel check
* Presence of fluorescent date of novel check
* Presence of static quick barcode

Because these security features have not been designed into any check before we believe this work is new in providing a safety measure, and will be provided to help with advanced levels of security, which will help workers in the field of the questioned document examination for detecting the forging crime through specifying the authorized date and position of printed documents from MIC.

## 3 Conclusion

In this study, we investigated a new methodology to date the valuable documents (checks and receipt). The approach used was focused on extracting the coded dots matrices (MIC) from color laser printers. For dating the print process, in a binary system for base 2 (0, 1) was used, while novel invisible UV inks based on a pyrazoline derivative were used for printing the date and security information. This approach was succeeded in presenting a new application of fluorescence‐active compounds for dating and securing valuable documents with an advanced level of security.

## 4 Experimental Section

*Materials—Paper Sample*: Durable paper sheets known as Bible paper (45 g m^−2^) were used as substrate for printing the valuable documents. This type was made from 25% cotton and linen in combination with chemical wood pulp.

*Materials—Polyvinyl Alcohol*: PVA (Mowiol grade 28–99) is a product of Hoechst Co. (Germany), with the following specifications: degree of saponification: 99.4%, viscosity of 4% aqueous solution: 28 cP, ester no.: 8 mg KOH per g, residual acetyl: 0.5, and ash: 0.5%.

*Materials—Fluorescence Active Compounds*: Four pyrazoline derivatives were synthesized^21^ and applied in UV ink to print the paper substrate. The methods of synthesizing these fluorescence compounds were summarized in the following scheme. These fluorescence compounds were selected from 21 compounds previously synthesized and have different fluorescence quantum yield (*ф*s). The choice of these fluorescence compounds, numbered 7–10, was based on their quantum yield values (*ф*s), fluorescence intensity of treated paper sheets and it mostly had positive effect on quality number of paper sheets.^21^

The general method for synthesizing 4,5‐dihydro‐1H‐pyrazoles (general procedure) is as follows

**Scheme 1:**
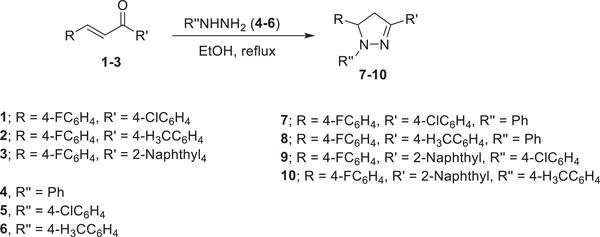
Synthetic route of pyrazoline fluorescence active compounds (7–10).

1,3,5‐Triaryl‐4,5‐dihydro‐1H‐pyrazoles compounds 7–10, were synthesized through a cyclocondensation reaction of equimolar amounts of 1,3‐diaryl‐2‐propen‐1‐ones 1–3 with aryl hydrazines 4–6 in refluxing ethanol. The solid separated upon storing the reaction mixture at room temperature overnight and was collected and crystallized from a suitable solvent, affording the corresponding pyrazolines 7–10. The obtained yields are in the range of 69% to 89% and are given in **Scheme** **1**.

Melting points were recorded on a Stuart SMP3 melting point apparatus. IR spectra (KBr) were recorded on a Shimadzu FT‐IR 8400S spectrophotometer. ^1^H‐NMR spectra were recorded on a Varian MERCURY 300 (300 MHz) and Bruker Ascend 400/R (400 MHz) spectrometers. 13C‐NMR spectra were recorded on a Bruker Ascend 400/R (100 MHz) spectrometer. Compounds 7–10 were prepared according to the reported procedures. UV spectra were recorded on a Shimadzu UV‐1800 spectrophotometer. Emission spectra were determined on LUMINA fluorescence spectrometer.

* 3‐(4‐Chlorophenyl)‐5‐(4‐fluorophenyl)‐1‐phenyl‐4,5‐dihydro‐1H‐pyrazole (7)

This compound was obtained from the reaction of 1 and 4 over 6 h. The almost colorless microcrystals were obtained from *n*‐butanol, mp 156–158 °C, yield 89%. IR ν (cm^−1^): 1589, 1558, 1504, and 1493. ^1^H‐NMR δ (ppm): (300 MHz) 3.08 (dd, *J* = 7.5, 17.1 Hz, 1H, upfield H of pyrazolinyl *H_2_C*‐4), 3.81 (dd, *J* = 12.5, 17.0 Hz, 1H, downfield H of pyrazolinyl *H_2_C*‐4), 5.28 (dd, *J* = 7.2, 12.3 Hz, 1H, pyrazolinyl *HC*‐5), 6.78‐6.85 (m, 1H, arom. H), 7.01–7.43 (m, 10H, arom. H), 7.65 (d, *J* = 8.7 Hz, 2H, arom. H). ^13^C‐NMR δ (ppm): (100 MHz) 43.4 (pyrazolinyl H_2_ *C*‐4), 64.0 (pyrazolinyl H*C*‐5), 113.5, 116.0, 116.2, 119.5, 125.3, 126.9, 127.1, 127.5, 127.6, 128.8, 128.9, 129.0, 129.1, 131.2, 134.4, 138.0, 138.1, 144.5, 145.6, 161.0, and 163.4 (arom. *C*). Elemental analysis: C_21_H_16_ClFN_2_ required C, 71.90; H, 4.60; N, 7.99, found C, 72.09; H, 4.71; N, 8.07.

* 5‐(4‐Fluorophenyl)‐3‐(4‐methylphenyl)‐1‐phenyl‐4,5‐dihydro‐1H‐pyrazole (8)

This compound was obtained from the reaction of 2 and 4 over 9 h. Pale yellow microcrystals were obtained from *n*‐butanol, mp 147–149 °C, yield 82% (1.35 g). IR ν (cm^−1^): 1597, 1551, and 1497. ^1^H‐NMR δ (ppm): (300 MHz) 2.39 (s, 3H, CH_3_), 3.10 (dd, *J* = 7.4, 17.0 Hz, 1H, upfield H of pyrazolinyl H_2_C‐4), 3.83 (dd, *J* = 12.5, 17.0 Hz, 1H, downfield H of pyrazolinyl H_2_C‐4), 5.24 (dd, *J* = 7.2, 12.3 Hz, 1H, pyrazolinyl HC‐5), 6.79–7.64 (m, 13H, arom. H). ^13^C‐NMR δ (ppm): (100 MHz) 21.4 (CH_3_), 43.7 (pyrazolinyl H_2_C‐4), 63.8 (pyrazolinyl HC‐5), 113.4, 115.9, 116.1, 119.2, 125.8, 127.5, 127.6, 129.0, 129.3, 129.9, 138.4, 138.5, 138.8, 144.9, 145.0, 160.9, and 163.4 (arom. C). Elemental analysis: C_22_H_19_FN_2_ required C, 79.97; H, 5.80; N, 8.48, found C, 80.04; H, 5.92; N, 8.64.

* 1‐(4‐Chlorophenyl)‐5‐(4‐fluorophenyl)‐3‐(2‐naphthyl)‐4,5‐dihydro‐1H‐pyrazole (9)

This compound was obtained from the reaction of 3 and 5 over 8 h. The pale yellow microcrystals were obtained from *n*‐butanol, mp 184–186 °C, yield 80% (1.60 g). IR ν (cm^−1^): 1595, 1495, and 1439. ^1^H‐NMR δ (ppm): (400 MHz) 3.25 (dd, *J* = 7.0, 17.0 Hz, 1H, upfield H of pyrazolinyl H_2_C‐4), 3.93 (dd, *J* = 12.3, 17.0 Hz, 1H, downfield H of pyrazolinyl H_2_C‐4), 5.26 (dd, *J* = 7.0, 12.2 Hz, 1H, pyrazolinyl HC‐5), 7.03–7.08 (m, 4H, arom. H), 7.16–7.19 (m, 2H, arom. H), 7.28–7.32 (m, 2H, arom. H), 7.50–7.53 (m, 2H, arom. H), 7.81–7.88 (m, 4H, arom. H), 8.17 (dd, *J* = 1.6, 8.6 Hz, 1H, arom. H). ^13^C‐NMR δ (ppm): (100 MHz) 43.6 (pyrazolinyl H_2_C‐4), 63.9 (pyrazolinyl H*C*‐5), 114.6, 116.1, 116.3, 123.4, 124.2, 125.4, 126.4, 126.58, 126.59, 127.5, 127.6, 127.9, 128.1, 128.3, 128.9, 129.2, 130.0, 133.3, 133.6, 137.7, 137.8, 143.1, 147.4, 161.0, and 163.5 (arom. C). Elemental analysis: C_25_H_18_ClFN_2_ required C, 74.90; H, 4.53; N, 6.99, found C, 75.12; H, 4.71; N, 7.18.

* 5‐(4‐Fluorophenyl)‐1‐(4‐methylphenyl)‐3‐(2‐naphthyl)‐4,5‐dihydro‐1H‐pyrazole (10)

This compound was obtained from the reaction of 3 and 6 over 6 h. The pale yellow microcrystals were obtained from *n*‐butanol, mp 188–189 °C, yield 84%. IR ν (cm^−1^): 1602, 1558, 1516, and 1504. ^1^H‐NMR δ (ppm): (400 MHz) 2.28 (s, 3H, CH_3_), 3.25 (dd, *J* = 7.4, 16.9 Hz, 1H, upfield H of pyrazolinyl H_2_C‐4), 3.91–3.99 (m, 1H, downfield H of pyrazolinyl H_2_C‐4), 5.27–5.32 (m, 1H, pyrazolinyl HC‐5), 7.04–7.08 (m, 6H, arom. H), 7.28–7.36 (m, 2H, arom. H), 7.48–7.51 (m, 2H, arom. H), 7.81–7.88 (m, 4H, arom. H), 8.19 (d, *J* = 8.7 Hz, 1H, arom. H). ^13^C‐NMR δ (ppm): (100 MHz) 20.5 (CH_3_), 43.5 (pyrazolinyl H_2_C‐4), 64.2 (pyrazolinyl HC‐5), 113.6, 115.9, 116.1, 123.5, 125.0, 125.4, 126.3, 126.5, 127.6, 127.7, 127.8, 128.1, 128.2, 128.7, 129.5, 129.7, 130.4, 133.3, 133.4, 138.39, 138.42, 142.5, 146.4, 160.9, and 163.4 (arom. C). Elemental analysis: C_26_H_21_FN_2_ required C, 82.08; H, 5.56; N, 7.36, found C, 82.24; H, 5.67; N, 7.22.

These synthesized heterocyclic compounds have fluorescence behavior, both in chloroform solution or after surface treating the paper sheets, as reported in Table 1 and 2. UV spectra were recorded on a Shimadzu UV‐1800 spectrophotometer. Emission spectra were determined on LUMINA fluorescence spectrometer.

*UV Ink Preparation*: The formulation of UV ink used in printing the security issues is as follows:

0.5 g polyvinyl alcohol, 0.1 g active fluorescence compound (7, 8, 9, and 10), 30 mL bidistilled water and 2 mL ethanol. First, polyvinyl alcohol was dissolved in 30 mL of hot distilled water with stirring to complete dissolution and formed a very clear, diluted solution. Then, 0.1 g of fluorescence compound (7, 8, 9, or 10) was added after cooling in an ice bath with 2 mL ethanol and stirred until complete dissolution occurred.

*Tools for Printing the Security Information*: Sirdas print dater 5810, numerator, and stamp print design were purchased from the library. The rubber printing stamp was designed for the special purpose of UV printing. The check and receipt were designed with Microsoft Word 2016.

*Tools for Printing the Security Information—Printing Techniques*: In our application, we selected the Xerox brand for color laser printers. The reason for this selection was based on the authorized date. All color laser printers used in this application are listed in **Table** **3**. Adobe Photoshop CC 2015 was applied, as nondestructive techniques for extraction the coded dots matrixes.

**Table 3:** Exhibiting the printers' models with the authorized date printed

No.	Printer model	Authorized coded date	UV printed date
1	Xerox Phaser 6500	15 JAN 2017	15 JAN 2017
2	Xerox WorkCentre 7665	05 JUN 2017	05 JUN 2017
3	Xerox WorkCentre 7675	07 JUN 2017	07 JUN 2017
4	Xerox WorkCentre 7132	12 JUN 2017	12 JUN 2017
5	Xerox DocuColor 250	22 AUG 2017	22 AUG 2017

## Conflict of Interest

The authors declare no conflict of interest.
